# Using the Zebrafish Lateral Line to Understand the Roles of Mitochondria in Sensorineural Hearing Loss

**DOI:** 10.3389/fcell.2020.628712

**Published:** 2021-02-05

**Authors:** Melanie Holmgren, Lavinia Sheets

**Affiliations:** ^1^Department of Otolaryngology, Washington University School of Medicine, St. Louis, MO, United States; ^2^Department of Developmental Biology, Washington University School of Medicine, St. Louis, MO, United States

**Keywords:** mitochondria, hearing loss, zebrafish, hair cell, lateral line

## Abstract

Hair cells are the mechanosensory receptors of the inner ear and can be damaged by noise, aging, and ototoxic drugs. This damage often results in permanent sensorineural hearing loss. Hair cells have high energy demands and rely on mitochondria to produce ATP as well as contribute to intracellular calcium homeostasis. In addition to generating ATP, mitochondria produce reactive oxygen species, which can lead to oxidative stress, and regulate cell death pathways. Zebrafish lateral-line hair cells are structurally and functionally analogous to cochlear hair cells but are optically and pharmacologically accessible within an intact specimen, making the zebrafish a good model in which to study hair-cell mitochondrial activity. Moreover, the ease of genetic manipulation of zebrafish embryos allows for the study of mutations implicated in human deafness, as well as the generation of transgenic models to visualize mitochondrial calcium transients and mitochondrial activity in live organisms. Studies of the zebrafish lateral line have shown that variations in mitochondrial activity can predict hair-cell susceptibility to damage by aminoglycosides or noise exposure. In addition, antioxidants have been shown to protect against noise trauma and ototoxic drug–induced hair-cell death. In this review, we discuss the tools and findings of recent investigations into zebrafish hair-cell mitochondria and their involvement in cellular processes, both under homeostatic conditions and in response to noise or ototoxic drugs. The zebrafish lateral line is a valuable model in which to study the roles of mitochondria in hair-cell pathologies and to develop therapeutic strategies to prevent sensorineural hearing loss in humans.

## Introduction

Hair cells are the mechanosensory receptors for hearing and balance in the inner ear. They convert vibrational stimuli into electrical signals sent to the brain. At their apical surfaces, hair cells have rows of actin-rich stereocilia (McGrath et al., 2017). Mechanical stimuli such as sound deflect these stereocilia, leading to opening of mechanotransduction (MET) channels and graded depolarization of the hair cell, a process that is modulated by Ca^2+^ (Mammano et al., 2007). This depolarization opens the voltage-dependent L-type Ca^2+^ channels Ca_V_1.3 localized at the basolateral membrane of hair cells, which drives synaptic vesicle fusion and release of glutamate onto auditory nerve fibers (Fettiplace, 2017).

MET and synaptic transmission are energy-demanding processes, and hair cells rely on mitochondria to generate ATP through oxidative phosphorylation to meet these high metabolic demands (Puschner and Schacht, 1997). One consequence of mitochondrial oxidative phosphorylation is the production of ROS. While low levels of ROS are important for maintaining homeostatic processes by acting as signaling molecules in intracellular pathways, high ROS levels can damage cells by oxidation of macromolecules (Collins et al., 2012). Damage by mitochondrial ROS has previously been linked to aging, although the role of ROS in aging processes is not completely understood (Liochev, 2013). Mitochondria also contribute to the homeostatic control of intracellular calcium. In hair cells, mitochondria directly regulate subcellular Ca^2+^ concentrations by uptake through the mitochondrial Ca^2+^ uniporter (Matlib et al., 1998; Wong et al., 2019). Mitochondria also indirectly regulate intracellular Ca^2+^ clearance by providing energy to fuel ATP-dependent Ca^2+^ pumps (PMCA) at the plasma membrane (Zenisek and Matthews, 2000). Ca^2+^ signaling is complex and is involved in a wide array of cellular processes and thus must be tightly regulated (Berridge et al., 2003). Mitochondria work in conjunction with the endoplasmic reticulum (ER) to buffer intracellular Ca^2+^ by sequestering it into these subcellular domains (Rizzuto and Pozzan, 2006; Rizzuto et al., 2009). Cytosolic Ca^2+^ is also buffered by proteins such as parvalbumin and calbindin-D28k (Hackney et al., 2005). As Ca^2+^ regulates both MET currents and synaptic vesicle release at opposite sides of the hair cell, this local Ca^2+^ regulation is critical for proper hair-cell function.

Mitochondria are important mediators in various cell death pathways, including apoptosis, necrosis, and autophagy. There are two distinct apoptosis signaling pathways; both pathways rely on a group of cysteine proteases called caspases, which cleave hundreds of proteins in apoptosis. In the cell-extrinsic apoptosis signaling pathway, cell death is triggered by ligands binding to cell-surface death receptors, resulting in formation of the death-inducing signaling complex (DISC) and activation of caspase-8 and caspase-10 (Ashkenazi, 2002). By contrast, in cell-intrinsic apoptosis signaling pathways, activation of pro-apoptotic Bcl-2 family proteins drives mitochondrial outer membrane permeabilization and triggers mitochondrial release of pro-apoptotic factors such as cytochrome *c*, which results in formation of the apoptosome and subsequent activation of caspase-9 (Liu et al., 1996; Du et al., 2000; Bock and Tait, 2020). These two pathways converge upon activation of the executioner caspases, caspase-3 and−7 (Bock and Tait, 2020). Necrosis and necroptosis, or programmed necrosis, are cell death pathways distinct from apoptosis and are characterized by mitochondrial swelling and impaired mitochondrial function. However, unlike apoptosis, cells that have been largely depleted of mitochondria are not resistant to necroptosis, suggesting that mitochondria are not critical for this cell death pathway (Tait et al., 2013). Autophagy, a lysosomal degradation process that can lead to cell death, has also been shown to be regulated by mitochondrial ROS (Chen et al., 2009).

Because the mitochondria play such critical roles in many cellular processes, they are increasingly being studied in the context of neural diseases, including sensorineural hearing loss (Johri and Beal, 2012; Bottger and Schacht, 2013). Hearing loss affects 23% of Americans over age 12 and has been associated with a variety of environmental causes (Goman and Lin, 2016). Acquired sensorineural hearing loss is caused by damage to hair cells and/or innervating afferent nerves. Hair cells can be damaged by excessive noise or by ototoxic drugs including aminoglycoside antibiotics and platinum-based chemotherapy drugs such as cisplatin (Lanvers-Kaminsky et al., 2017). Gradual loss of hearing, due to loss of hair cells, is also a common problem linked to aging (Schuknecht and Gacek, 1993). Because hearing loss is so prevalent and hair cell loss is permanent, there is a need to understand mechanisms of damage and to develop preventative and restorative therapies.

**Figure 1:**
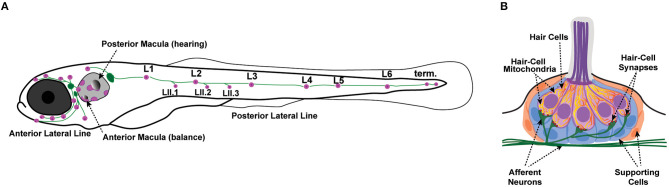
Zebrafish lateral-line neuromasts. **(A)** Schematic depicts a larval zebrafish. Pink patches indicate the location of hair cells in the inner ear required for hearing and balance, as well as hair cells in the lateral-line system. Green patches represent the location of the anterior and posterior lateral-line ganglia. The cell bodies of neurons in these ganglia project to and innervate hair cells in the lateral line. **(B)** A side view of the anatomy of a single lateral-line neuromast. Hair cells (pink) are surrounded by supporting cells (internal, blue and peripheral, orange) and innervated by both afferent (green) and efferent neurons. Mechanosensory hair bundles (purple) at the apex of hair cells project out into the water to detect local water flow. Mitochondria (yellow, orange) make up dynamic tubular networks within hair cells. Adapted from Kindt and Sheets (2018).

Zebrafish have emerged as a powerful model to investigate hearing and balance disorders. Zebrafish became established as a model organism for studying hair-cell development and function due to identification of numerous conserved genes involved in hearing and balance (Nicolson, 2017). In addition to their inner ears, which are required for hearing and balance, zebrafish also have hair cells in their lateral line organs. Unlike the sensory organs of the inner ear, lateral-line organs are located along the surface of the body, are used to detect local water currents (Dijkgraaf, 1963), and mediate behaviors such as the escape response (to avoid predation) and rheotaxis (counterflow swimming) (Olszewski et al., 2012; Suli et al., 2012; Stewart et al., 2013; Olive et al., 2016). The lateral line is made up of clusters of hair cells and supporting cells called neuromasts, which are innervated by afferent and efferent neurons (Figure 1) (Metcalfe et al., 1985; Raible and Kruse, 2000). The lateral line system in particular has become increasingly popular for studying hair-cell biology due to the optical and pharmacological accessibility of the neuromasts. In addition, in contrast to hair cells in the mammalian inner ear, fish hair cells in the ear and lateral line organs can regenerate after damage (Balak et al., 1990; Lombarte et al., 1993). Moreover, the generation of numerous transgenic fish lines expressing genetically encoded fluorescent reporters in hair cells offers the ability to visualize cellular and subcellular dynamics *in vivo* (Esterberg et al., 2014; Kindt and Sheets, 2018; Pickett et al., 2018). The zebrafish lateral line is thus a useful model system in which to study hair-cell biology and has been used to elucidate the roles of mitochondria in hair-cell pathologies and in homeostasis.

## Identifying Roles of Mitochondria in the Lateral Line Under Homeostatic Conditions

In addition to generating ATP and contributing to the spatial regulation of calcium within the cell, recent work has established novel roles for mitochondria in the development and maintenance of hair-cell synapses. Hair cells contain specialized electron-dense presynaptic structures, known as synaptic ribbons, that tether synaptic vesicles at the active zone and correspond with presynaptic clusters of voltage-gated L-type calcium channels (Ca_V_1.3) (Frank et al., 2010; Sheets et al., 2011). Vesicle fusion occurs at hair-cell ribbon synapses upon influx of Ca^2+^ through Ca_V_1.3 (Brandt et al., 2003). It has been demonstrated in mammals that spontaneous Ca^2+^ influx through Ca_V_1.3 occurs in developing hair cells (Marcotti et al., 2003; Tritsch et al., 2007, 2010; Eckrich et al., 2018). Previous work in zebrafish revealed a role for presynaptic Ca^2+^ influx in modulating synaptic ribbon size within developing lateral-line hair cells; enlarged ribbons were observed in *cav1.3a* mutant hair cells, or in hair cells exposed to the L-type Ca^2+^ channel blocker isradipine (Sheets et al., 2012), while treatment with the L-type Ca^2+^ channel agonist Bay K8644 led to decreased ribbon size. A recent study further defined the role of mitochondria in this process (Wong et al., 2019). Spontaneous presynaptic Ca^2+^ influx was observed in developing zebrafish lateral-line hair cells and, in response to this influx, mitochondria localized near synaptic ribbons showed Ca^2+^ uptake, a process dependent on both Ca_V_1.3 and the mitochondrial Ca^2+^ uniporter (MCU) (Wong et al., 2019). Blocking mitochondrial Ca^2+^ uptake with the MCU inhibitor Ru360 led to increased synaptic ribbon size in developing hair cells, demonstrating a role of mitochondrial Ca^2+^ signaling in ribbon formation during development.

Mitochondrial Ca^2+^ uptake likely regulates synaptic ribbon size by influencing NAD^+^/NADH redox (Jensen-Smith et al., 2012). The major structural component of synaptic ribbons is a protein called RIBEYE (Schmitz et al., 2000; Sheets et al., 2011; Lv et al., 2016). RIBEYE contains a unique A-domain and a B-domain which is nearly identical to the transcriptional repressor protein CtBP2, and each domain contains binding sites that regulate the formation of RIBEYE aggregates. Notably, RIBEYE B-domain contains an NAD(H) binding site, and it has been shown *in vivo* that NAD(H) inhibits heteromeric interactions between RIBEYE A- and B-domains (Magupalli et al., 2008). In zebrafish hair cells, blocking mitochondrial Ca^2+^ uptake with Ru360 or inhibiting Ca_V_1.3 with isradipine resulted in increased NAD^+^/NADH ratio (Wong et al., 2019). Exogenously manipulating NAD^+^/NADH also altered presynaptic ribbon sizes in developing hair cells, such that treatment with NAD^+^ led to enlarged ribbons, while treatment with NADH decreased ribbon size. Cumulatively, these data support that spontaneous hair-cell activity and mitochondrial Ca^2+^ accumulation regulates NAD^+^/NADH redox, which in turn modulates synaptic ribbon assembly in developing hair cells.

In addition to playing a key role in development of hair-cell synapses, mitochondrial activity also appears to be critical for synaptic maintenance. In mature hair cells, activity evoked mitochondrial Ca^2+^ uptake functions to sustain presynaptic Ca^2+^ responses and maintain synapse integrity. Blocking mitochondrial Ca^2+^ uptake by treating with Ru360 or the voltage-dependent anion channel (VDAC) inhibitor TRO 19622 impaired presynaptic Ca^2+^ signals (Wong et al., 2019). In addition, partially blocking mitochondrial Ca^2+^ uptake with a low dose of Ru360 led to a high density of presynaptic Ca_V_1.3 channel clusters, while completely blocking MCU with a high dose of Ru360 led to increased synaptic ribbon size and a reduction in the number of synapses per hair cell. These data establish a role of presynaptic Ca^2+^ signaling in the maintenance of hair-cell ribbon synapses. It is likely that this process occurs via a mitochondrial mechanism, as inhibition of mitochondrial Ca^2+^ uptake with Ru360 did not affect cytosolic Ca^2+^ concentrations, while modulation of Ca_V_1.3 with isradipine or Bay K8644 resulted in changes in mitochondrial Ca^2+^ levels (Wong et al., 2019).

In conjunction with the mitochondrion, the endoplasmic reticulum (ER) is also a critical regulator of Ca^2+^ signaling within the cell (Schwarz and Blower, 2016). These two organelles are associated with mitochondrial associated membranes (MAMs), and Ca^2+^ transfer between them has been implicated in a wide range of cellular processes, including bioenergetics and cell death (Vance, 1990; Rizzuto et al., 2009; Bravo et al., 2012; Giorgi et al., 2012; Grimm, 2012). In zebrafish lateral-line hair cells, it has been demonstrated that mitochondria buffer Ca^2+^ released from the ER (Esterberg et al., 2014). Using a mitochondrial matrix-targeted Ca^2+^ indicator GCaMP3 (mitoGCaMP3), they observed increased fluorescence, indicative of increased local Ca^2+^ concentration, upon activation of inositol trisphosphate receptors (IP_3_Rs) with adenophostin A, and decreased GCaMP fluorescence upon inhibition of IP_3_Rs with xestospongin C or by blocking the MCU with Ru360. IP_3_Rs in the ER are involved in Ca^2+^ release into the cytosol and are coupled with mitochondrial VDAC in MAMs (Szabadkai et al., 2006). Thus, the observed changes in mitochondrial Ca^2+^ upon modulation of IP_3_Rs support the flow of Ca^2+^ from the ER to the mitochondria (Esterberg et al., 2014). It has been previously suggested that Ca^2+^ originating in the ER is first transferred to the cytosol before being taken up by mitochondria (Patergnani et al., 2011). To simultaneously monitor cytoplasmic and mitochondrial Ca^2+^ dynamics in the same cell, Esterberg et al. (2014) combined mitoGCaMP3 with the red cytosolic Ca^2+^ indicator RGECO and found that transient increases in cytosolic RGECO fluorescence corresponded with mitochondrial GCaMP3 increases. Upon ER modulation with adenophostin A, they observed increases in both cytosolic and mitochondrial Ca^2+^, but upon treatment with thapsigargin, only an increase in mitochondrial Ca^2+^ was observed. Further, by using local uncaging of photolabile EGTA preloaded with Ca^2+^ (caEGTA) to transiently elevate intracellular Ca^2+^ levels, they observed increased mitochondrial Ca^2+^ uptake that corresponded with an increase in mitochondrial transmembrane potential (ΔΨ_m_), suggesting that even transient increases in mitochondrial Ca^2+^ can affect mitochondrial activity in hair cells. Cumulatively, these results show that under non-pathological conditions mitochondria take up Ca^2+^ released from the ER and that changes in mitochondrial Ca^2+^ can alter mitochondrial activity.

**Figure 2:**
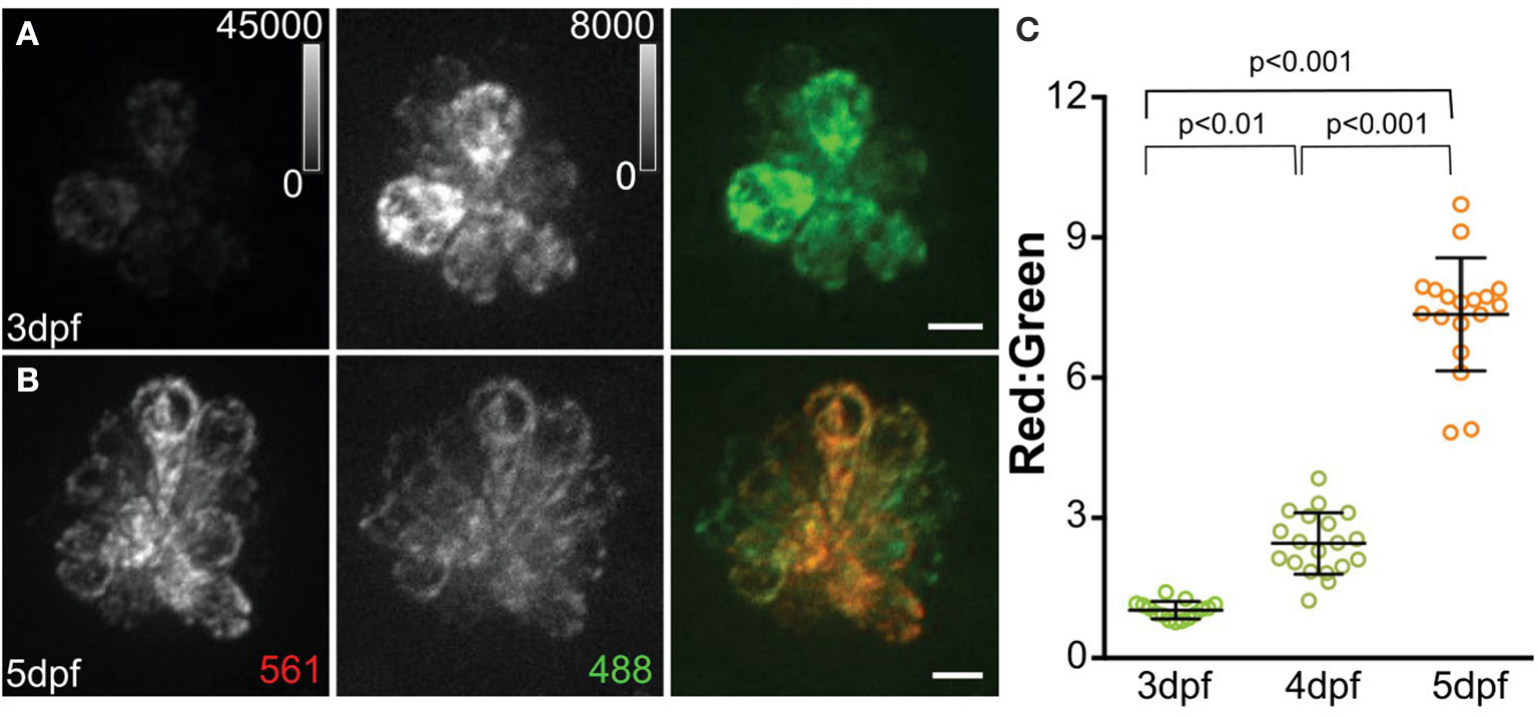
MitoTimer fluorescence in hair cells. **(A, B)** Maximum-intensity projections of hair cells from *Tg[myo6b:mitoTimer]*^*w*208^ fish at 3dpf **(A)** and 5dpf **(B)**. **(C)** Mean mitoTimer fluorescence plotted as ratio of red:green at 3, 4, and 5 dpf. Scale bar, 5 μm. Reproduced with permission from Pickett et al. (2018).

A recent study defined the short- and long-term consequences of evoked hair-cell activity on mitochondrial function. Using the same cytosolic and mitochondrial Ca^2+^ indicators described earlier, Pickett et al. (2018) observed increases in both cytosolic and mitochondrial Ca^2+^ in response to acute stimulation of hair cells, i.e., directional displacement of hair-cell stereocilia via a waterjet. Notably, the two evoked Ca^2+^ signals showed different kinetics, with rapid onset and decay in cytosolic Ca^2+^ signal followed by a slower rise and longer decay in mitochondrial Ca^2+^ signal. Mutants lacking mechanotransduction showed a reduced mitochondrial transmembrane potential, indicating reduced mitochondrial activity in the absence of hair-cell activity. To define long-term consequences on mitochondrial function, they next examined how cumulative hair-cell activity influenced the state of mitochondria using MitoTimer, a genetically encoded indicator of mitochondrial oxidation. Newly synthesized MitoTimer fluoresces green, but irreversibly shifts to red upon dehydrogenization of the Tyr-67 residue (Figure 2). Sustained lateral-line hair-cell stimulation in larvae exposed to 24 h of water currents generated by orbital rotation resulted in significantly increased MitoTimer fluorescence ratio that corresponded with increased hair-cell ROS, as measured with cellROX (a probe for oxidative stress; Table 1), and was dependent on hair-cell MET. Increased MitoTimer fluorescence also occurred with age; older hair cells had a higher red:green MitoTimer ratio compared with younger hair cells. These data support that hair-cell activity influences cumulative mitochondrial activity and hair-cell oxidation.

**Table 1:** Drugs, vital dyes, and indicators used to study hair-cell mitochondrial biology.

**Drug or indicator**	**Function**	**Dosage or working concentration**	**References**
2-{4-(dimethylamino)styryl}-*N*-ethylpyridinium iodide (DASPEI)	Mitochondrial stain	0.005%	Owens et al., 2007; Hong et al., 2013; Song et al., 2014; Rah et al., 2015; Chang et al., 2016; Kruger et al., 2016; Hayward et al., 2019
MitoTracker Green FM	Mitochondrial mass indicator	25–100 nM	Owens et al., 2007; Kuang et al., 2017; Zhou et al., 2019
FM1-43	Mechanotransduction-dependent dye	1–2.25 μM	Kruger et al., 2016; Stawicki et al., 2016; Kuang et al., 2017
Tetremethylrhodamine ethyl ester (TMRE)	Mitochondrial membrane potential indicator	1–25 nM	Esterberg et al., 2013, 2014, 2016; Alassaf et al., 2019
JC-1	Ratiometric mitochondrial membrane potential indicator	1.5 μM	Pickett et al., 2018
MitoTracker Red CMXRos	Mitochondrial membrane potential indicator	25 nM	Owens et al., 2007
3-Diethyloxacarbocyanine iodide [DiOC2(3)]	Mitochondrial membrane potential indicator	0.1 μM	Kuang et al., 2017
Yo-Pro1	Nuclear stain	3 μM	Hayward et al., 2019
cellROX Green	ROS indicator	2–2.5 μM	Esterberg et al., 2016; Pickett et al., 2018
cellROX Deep Red	ROS indicator	10 μM	Alassaf et al., 2019
H_2_DCFDA	ROS indicator	10 μM	Hirose et al., 2016
mitoSOX	Mitochondrial superoxide indicator	1 μM	Esterberg et al., 2016; Alassaf et al., 2019)
mitoTEMPO	Superoxide scavenger; TPP+-modified version of TEMPOL	10–100 μM	Esterberg et al., 2016; Alassaf et al., 2019
TEMPOL	Superoxide scavenger	50 μM	Esterberg et al., 2016
*o*-Nitrophenyl EGTA (NP-caged EGTA)	Photolabile Ca^2+^ chelator; used to deliver Ca^2+^ upon UV exposure	25 pmol (injected at 1-cell stage)	Esterberg et al., 2013, 2014
Diazo2	Ca^2+^ chelator	25 pmol (injected at 1-cell stage)	Esterberg et al., 2013
Ionomycin	Ca^2+^ ionophore	5 μM	Esterberg et al., 2013
Cyclosporin A (CsA)	Inhibitor of cyclophilin D	200 nM	Esterberg et al., 2016
Carbonyl cyanide 4-(trifluoromethoxy) phenylhydrazone (FCCP)	Uncoupler of mitochondrial oxidative phosphorylation	100 nM−10 μM	Kim M. J et al., 2008; Zhou et al., 2019
Antimycin A	Inhibitor of mitochondrial electron transport chain	100–500 pM	Alassaf et al., 2019
Ru360	Antagonist of mitochondrial Ca^2+^ uniporter (MCU)	500 nM−10 μM	Esterberg et al., 2016; Wong et al., 2019
TRO 19622	Antagonist of voltage-dependent anion channel (VDAC)	10 μM	Wong et al., 2019

## Mitochondria in Response to Aminoglycoside Ototoxicity

Aminoglycoside antibiotics, such as neomycin and gentamicin, are widely used to treat Gram-negative bacterial infections, such as tuberculosis (Forge and Schacht, 2000). These drugs also cause hearing loss in up to 20% of patients who take them, and this hearing loss is due to aminoglycoside-mediated hair-cell death (Xie et al., 2011). While it is known that aminoglycosides function by binding to ribosomal subunits and interfering with bacterial translation, the mechanisms underlying hair-cell death were still unclear (Borovinskaya et al., 2007, 2008). Further, there is evidence that different aminoglycosides kill hair cells by different pathways (Coffin et al., 2013b). There is thus a need to understand the various mechanisms of ototoxic damage by aminoglycosides to prevent loss of hair cells. In addition, it has been demonstrated that the mitochondrial ribosome closely resembles the prokaryotic ribosome, suggesting a role of mitochondria in the response to aminoglycosides (Lynch and Puglisi, 2001).

Neomycin has been widely used to study ototoxic effects in the zebrafish lateral line, and an early analysis of the hair-cell ultrastructure in response to neomycin demonstrated mitochondrial swelling that preceded other cellular phenotypes (Owens et al., 2007). More recent work has shown that a hair cell's history of mitochondrial activity can predict its susceptibility to neomycin-induced death, such that hair cells with more cumulative mitochondrial activity, including hair cells that are older, are more vulnerable to aminoglycoside-induced death (Pickett et al., 2018).

**Figure 3:**
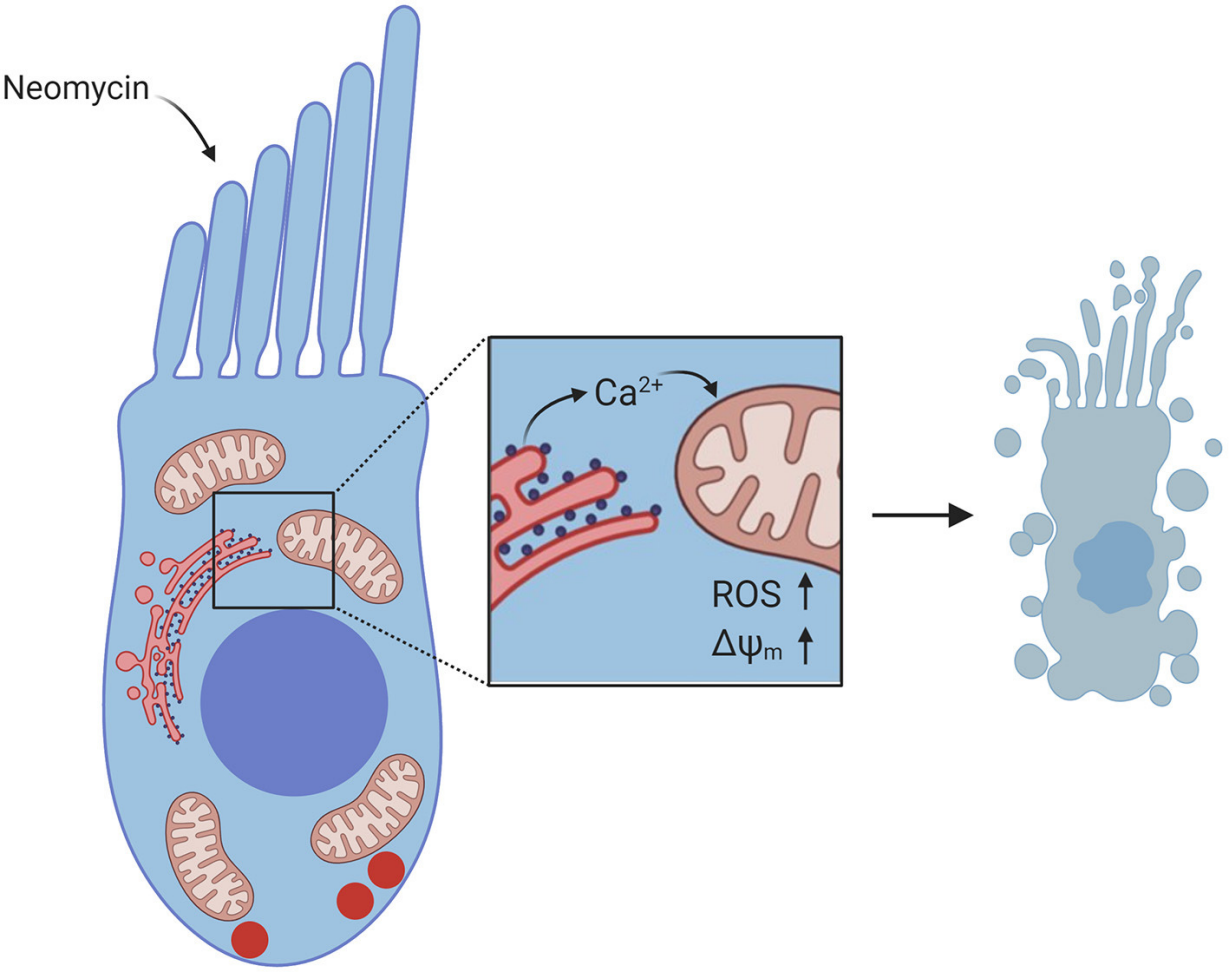
Diagram of Ca^2+^ signaling in hair cells exposed to neomycin. Neomycin enters the hair cell through MET channels at the apex. Inset shows flow of Ca^2+^ from the ER to the mitochondria, resulting in increased ROS levels and increased ΔΨ_m_, which leads to hair-cell death. Created with BioRender.com.

As mitochondria play a critical role in regulating intracellular Ca^2+^, recent studies have sought to characterize mitochondrial Ca^2+^ dynamics in the context of neomycin-induced hair-cell damage. In dying hair cells exposed to neomycin, the mitochondrial potential (ΔΨ_m_) collapses, followed by a spike in cytosolic Ca^2+^ (Esterberg et al., 2013). Elevating intracellular Ca^2+^ by uncaging caEGTA resulted in hair-cell death and increased susceptibility to neomycin, while decreasing intracellular Ca^2+^ by uncaging the Ca^2+^ chelator diazo2 exhibited a protective effect. In addition, it has been observed that both cytosolic Ca^2+^ and mitochondrial Ca^2+^ levels spike in dying hair cells exposed to neomycin (Esterberg et al., 2014). Inducing Ca^2+^ release from the ER by treatment with thapsigargin or activating IP_3_Rs with adenophostin A increased neomycin-induced hair-cell death, while inhibiting IP_3_Rs with xestospongin C protected hair cells. Blocking mitochondrial Ca^2+^ uptake with Ru360 also protected hair cells, suggesting that the flow of Ca^2+^ from the ER to the mitochondria is a defining event in neomycin-induced hair-cell death. This Ca^2+^ transfer from the ER to the mitochondria drives increased mitochondrial hyperpolarization during hair-cell death (Figure 3). Further, modulating mitochondrial polarization altered hair-cell susceptibility to neomycin, such that increasing ΔΨ_m_ by treatment with cyclosporin A increased neomycin toxicity, while mitochondrial depolarization by treatment with FCCP protected hair cells from neomycin-induced death. These data provide a mechanism by which mitochondrial Ca^2+^ plays a key role in neomycin-induced hair-cell death.

While the mechanisms underlying neomycin-induced hair-cell death have been well researched, gentamicin ototoxicity has been relatively understudied in the zebrafish lateral line. It has been shown that the hair-cell signaling pathways activated in response to gentamicin and time course of hair-cell death are different from that of neomycin; it is thus important to understand the mechanisms of damage by different aminoglycosides to develop protective therapies (Owens et al., 2009; Coffin et al., 2013a,b; Wiedenhoft et al., 2017). While a requirement for caspases in aminoglycoside-induced hair-cell death has been established *in vivo* in chick and *in vitro* in mammalian inner ear cultures, a study in the zebrafish lateral line demonstrated that both neomycin and gentamicin induce hair-cell death by caspase-independent pathways, as treatment with the caspase inhibitor Z-VAD-Fmk did not confer protection against either aminoglycoside (Coffin et al., 2013b). One study defined the relative roles of p53 signaling in neomycin and gentamicin ototoxicity. In apoptosis, p53 rapidly translocates to the mitochondria, preceding other mitochondrial phenotypes such as changes in ΔΨ_m_ and release of cytochrome *c* (Marchenko et al., 2000). In the mitochondria, p53 directly interacts with Bcl-2 to block its anti-apoptotic activity and induces apoptosis by interacting with pro-apoptotic proteins such as Bax (Mihara et al., 2003; Chipuk et al., 2004; Deng et al., 2006). Inhibition of the pro-apoptotic protein Bax offered some protection against neomycin- but not gentamicin-induced hair-cell loss, suggesting a Bax-dependent cell death pathway is involved in neomycin ototoxicity. In contrast, the p53 inhibitor pifithrin-α (PFTα) offered protection against gentamicin-induced hair-cell death suggesting an alternative, p53-dependent pathway underlying gentamicin ototoxicity. This is underscored by the observation that stabilizing p53 by treating with nutlin-3a, an inhibitor of the p53 antagonist Mdm2, sensitized hair cells to toxicity from chronic gentamicin exposure (Coffin et al., 2013a). In the same study, they found that overexpression of Bcl2, a target of p53, robustly protected zebrafish against gentamicin-induced hair-cell loss, but that some hair-cell death occurred after the course of treatment, implying that delayed hair-cell death following continuous gentamicin treatment is independent of p53. Future studies profiling the pathways involved in aminoglycoside-induced hair-cell death and defining the roles of proteins such as p53 and Bax will be useful in identifying therapeutic targets to protect against aminoglycoside ototoxicity. In addition, it is important to consider the differences between model systems in response to aminoglycosides, such as the requirement for caspases.

While there are obvious differences between model organisms, such as the differences between zebrafish neuromasts and the mammalian cochlea, there may also be differences between strains of a single species. A recent study found that the Tupfel long-fin (TL) wild-type strain of zebrafish were less vulnerable to gentamicin-induced hair-cell loss than the AB^*^ strain of zebrafish (Wiedenhoft et al., 2017). Both of these strains of zebrafish are commonly used for a variety of studies, including studies of hair-cell biology. They observed that, while stabilizing p53 with nutlin-3a sensitized AB^*^ hair cells to gentamicin-induced death, nutlin-3a treatment did not affect the survival of TL hair cells exposed to gentamicin, suggesting there may be strain-specific differences in p53 signaling. It is thus important to also consider the strain of zebrafish in studies of hair-cell biology.

## Mitochondrial ROS Production and Antioxidant Protection Against Environmental Hair-Cell Damage

One consequence of oxidative phosphorylation is the generation of ROS. Mitochondrial ROS are generally produced in the form of superoxide or hydrogen peroxide due to oxidation of metabolic intermediates in the electron transport chain complexes I and III (Brand, 2016). Hydroxyl radicals can also be produced in the mitochondria by the Fenton reaction, in which iron compounds are reduced by superoxide (Thomas et al., 2009). Under homeostatic conditions, ROS production is tightly regulated. Under conditions of oxidative stress, however, cytosolic ROS can stimulate the further production of ROS, potentially leading to a cascade of oxidative damage to macromolecules within the cell (Kroller-Schon et al., 2014).

Mitochondrial ROS production may play a role in aminoglycoside ototoxicity. It has been shown that aminoglycosides bind to iron salts and stimulate the production of free radicals by Fenton chemistry (Priuska and Schacht, 1995; Kohanski et al., 2007). A study in zebrafish lateral line observed an increase in ROS levels, as measured by the indicator cellROX green and by the hydrogen peroxide biosensor HyPer, in dying hair cells upon neomycin exposure (Esterberg et al., 2016). The increased hydrogen peroxide level corresponded with increased ΔΨ_m_ and mitochondrial oxidation, as reflected by the indicator mitoSOX (Table 1). These observed phenotypes were driven by mitochondrial Ca^2+^ uptake; blocking entry of Ca^2+^ into the mitochondria with Ru360 reduced ROS levels and mitochondrial oxidation after neomycin exposure. Cumulatively, these data suggest that mitochondrial Ca^2+^ uptake is an event upstream of neomycin ototoxicity, with ROS playing an additional role.

Exogenous antioxidants have shown promising otoprotective effects in zebrafish lateral line and mammalian cochlear explants (Ton and Parng, 2005; Noack et al., 2017; Hur et al., 2018). However, this approach has had limited success in clinical trials, likely because different antioxidants act via distinct cellular targets and molecular pathways (Noack et al., 2017). While treatment with exogenous antioxidants or ROS scavengers is one potential approach to protect hair cells from ototoxicity, another strategy is to leverage endogenous mechanisms within cells to protect against damage by ROS. To guard against oxidative stress, organisms have developed endogenous defense mechanisms, including antioxidant enzymes such as catalase, glutathione peroxidase, superoxide dismutases, and heme oxygenase-1 (HO-1) (Mates, 2000). Expression of these antioxidant enzymes can be induced by activators of peroxisome proliferator-activated receptor-α (PPAR-α) (Toyama et al., 2004). Fenofibrate, one such PPAR-α agonist, offered modest protection against gentamicin-induced hair-cell loss in both rat cochlear explants and in zebrafish lateral-line neuromasts (Park et al., 2017). This protection required activity of HO-1, as inhibition of HO-1 by SnPPIX abolished these protective effects. Thus, stimulating endogenous antioxidant pathways could also provide some protection against aminoglycoside-induced damage. Another approach to protecting against aminoglycoside ototoxicity is the use of ROS scavengers. Treatment of zebrafish with NecroX-5, an ROS and RNS scavenger, offered modest protection against neomycin-induced damage (Song et al., 2014). NecroX-5 exposure partially rescued neomycin-induced phenotypes including hair-cell loss, hair-cell apoptosis, and damage to hair bundles and hair-cell mitochondria. Treatment with quercetin, another ROS scavenger which has been shown to have antioxidant properties *in vitro*, also protected against neomycin-induced hair-cell loss (Hirose et al., 2016). Quercetin also reduced ROS levels, as detected by H_2_DCFDA labeling (Table 1).

In mammals, it has been shown that ROS are generated in the cochlea after noise exposure, and that antioxidants administered before or after exposure can potentially ameliorate noise-induced damage (Yamane et al., 1995; Ohlemiller et al., 1999; Ohinata et al., 2000; Oishi and Schacht, 2011). A recent study sought to model severe noise damage in the zebrafish lateral line using an ultrasonic device to produce cavitation creating small localized shock waves, and found that exposure to this stimulus resulted in hair-cell death 48–72 h after exposure (Uribe et al., 2018). Treatment with the antioxidant d-methionine prevented this sonic-induced hair-cell loss, suggesting a role for oxidative stress in this model. Because zebrafish can be exposed to drugs by bath application, they are an optimal system in which to screen for protective or harmful drugs. By screening a redox library for compounds that protected against damage, glutathione, baicalein, d-α-tocopherylquinone, and ferulic acid ethylester were identified as protective agents (Uribe et al., 2018). As reducing oxidative stress gains attention as a strategy to prevent noise-induced hair-cell death, zebrafish models may provide information toward identifying effective protective therapeutic strategies.

It has been suggested, specifically in the context of aminoglycoside ototoxicity, that hair cells “find a way to die” such that inhibition of one death pathway will lead to the activation of other death pathways, and that it may be necessary to target multiple pathways to fully protect hair cells from ototoxins, such as by using drug cocktails or by using drugs that have multiple modes of action (Vandenabeele et al., 2006; Ou et al., 2012). It has been determined that aminoglycosides enter hair cells through MET channels, so one approach to protecting against aminoglycoside ototoxicity could be to block uptake through MET (Alharazneh et al., 2011; Hailey et al., 2017). In zebrafish, it has been shown that treatment with quinoline ring derivatives such as amsacrine, quinine, and mefloquine protects against aminoglycoside-induced hair-cell death by blocking uptake (Ou et al., 2012). Analogs of the quinoline ring derivative berbamine have also recently been shown to block uptake of aminoglycosides into hair cells; however, the degree of uptake block did not correlate with the magnitude of hair-cell protection from aminoglycosides, suggesting a second uptake-independent mechanism (Kruger et al., 2016; Hudson et al., 2020). Quinoline ring derivatives have been shown to have antioxidant properties in other systems (Detsi et al., 2007; Naik et al., 2009; Ghinet et al., 2012). It would be interesting to know whether these compounds also function as antioxidants in a zebrafish or mammalian model of ototoxicity, as it will be important to identify drugs that could target both aminoglycoside uptake and oxidative stress in hair cells as candidate treatments for the prevention of aminoglycoside-induced hearing loss in humans.

## Oxidative Stress and Cisplatin

Cisplatin is an anti-cancer chemotherapeutic drug that is commonly used to treat a number of different cancers. Notably, cisplatin treatment causes hearing loss in up to 80% of patients (Frisina et al., 2016). Cisplatin ototoxicity has been linked to ROS, in that ROS deplete cochlear tissue of antioxidants, leading to increased free radical production and subsequent lipid peroxidation (Rybak, 2007). In addition, cisplatin may induce hair-cell death, at least in part, by activating ROS-mediated cell death pathways in mitotically quiescent hair cells (Kim C. H et al., 2008). This mode of induction is in contrast to mitotically active cells, where cisplatin induces cell death through DNA damage and activation of apoptosis.

Similar to work with aminoglycosides, protection against cisplatin ototoxicity using antioxidants and ROS scavengers has been an intriguing topic of recent study. Epicatechin, a ROS scavenger derived from tea leaves, has been shown to protect zebrafish from cisplatin-induced lateral-line hair-cell loss (Kim C. H et al., 2008). Kenpaullone, an inhibitor of cyclin-dependent kinase 2, was shown to reduce ROS in cochlear explants and also protected against cisplatin-induced damage in the zebrafish lateral line (Teitz et al., 2018). Quercetin, similar to its effect on aminoglycoside ototoxicity, protected against cisplatin-induced hair-cell loss and mitochondrial damage (Lee et al., 2015). Finally, the free radical scavenger edaravone also showed a protective effect against cisplatin-induced hair-cell loss, mitochondrial damage, and damage to hair bundles (Hong et al., 2013). While it has been shown that cisplatin ototoxicity in the lateral line is both dose and time dependent (Ou et al., 2007), one notable deficit in the area of cisplatin ototoxicity research in zebrafish is a current lack of consistent cisplatin damage protocols, i.e., optimal dosage and time of exposure used across studies (Domarecka et al., 2020). As cisplatin acts as a cytotoxin through diverse cellular pathways, optimizing damage protocols in zebrafish will be required to determine the relative role of ROS accumulation in cisplatin ototoxicity.

## Using Genetic Tools to Study Zebrafish Hair-Cell Mitochondrial Biology

While acquired hearing loss can be caused by exposure to noise or ototoxic drugs, some hearing loss is inherited (Lenz and Avraham, 2011; Mahboubi et al., 2012). In addition, susceptibility to age-related, noise-induced, or aminoglycoside-induced hearing loss may also have a genetic component. Recent whole-genome sequencing and genome-wide association studies have identified genes implicated in acquired hearing loss (Zhao et al., 2013; Lavinsky et al., 2015; Vuckovic et al., 2018; Nagtegaal et al., 2019; Wells et al., 2019). Interestingly, mitochondrial mutations have also been associated with hearing loss susceptibility (Jing et al., 2015). Identifying and studying both mitochondrial genes and genes involved in regulating mitochondrial function will undoubtedly aid in developing protective therapies.

**Figure 4:**
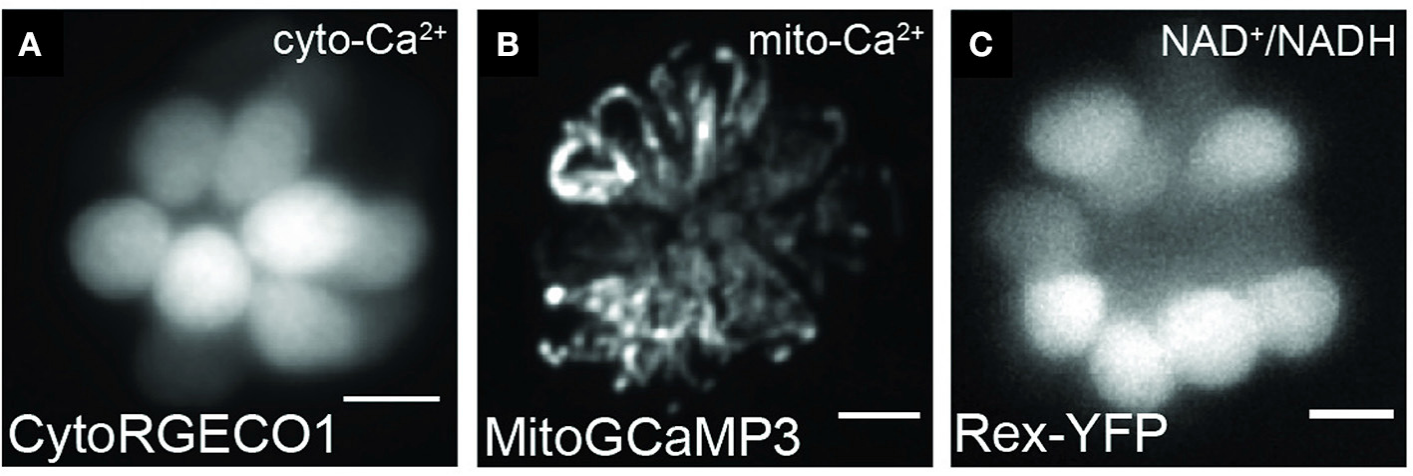
Representative images of hair cells expressing transgenes used to visualize mitochondrial dynamics. Live hair cells expressing RGECO1 **(A)**, MitoGCaMP3 **(B)**, and Rex-YFP **(C)**. Scale bar, 5 μm. Adapted with permission from Wong et al. (2019).

Zebrafish have been a popular model for genetic studies due to their high fecundity and ease of genetic manipulation. Forward genetic screens in zebrafish have proven useful for identifying genes required for hearing and balance as well as in pathways involved in aminoglycoside-induced death (Nicolson et al., 1998; Owens et al., 2008; Stawicki et al., 2016; Nicolson, 2017). Through reverse genetics, using tools such as CRISPR/Cas9, one can generate mutations in zebrafish as a way to study those implicated in hearing loss in humans (Liu et al., 2019). In addition to generating mutations to study gene function, transgenic fluorescent reporters can also be used to visualize cellular and subcellular structures and dynamics (Table 2; Figure 4).

**Table 2:** Transgenic zebrafish lines used to visualize and study hair-cell mitochondrial dynamics.

**Transgene**	**Description**	**References**
Tg[myo6b:cytoRGECO]	Hair-cell specific Ca^2+^ biosensor	Esterberg et al., 2014
Tg[myo6b:cytoGCaMP3]	Hair-cell specific Ca^2+^ biosensor	Esterberg et al., 2013
Tg[myo6b:mitoRGECO]	Mitochondrially localized Ca^2+^ biosensor in hair cells	Esterberg et al., 2014
Tg[myo6b:mitoGCaMP3]	Mitochondrially localized Ca^2+^ biosensor in hair cells	Esterberg et al., 2014
Tg[myo6b:erGCaMP3]	ER-localized Ca^2+^ biosensor in hair cells	Esterberg et al., 2014
Tg[myo6b:GCaMP6s-CAAX]	Membrane-localized Ca^2+^ biosensor in hair cells	Jiang et al., 2017
Tg[myo6b:HyPer]	Hair-cell specific hydrogen peroxide biosensor	Esterberg et al., 2016
Tg[myo6b:Rex-YFP]	NAD(H) redox fluorescent indicator in hair cells	Wong et al., 2019
Tg[myo6b:mitoTimer]	Mitochondrially localized fluorescent indicator of mitochondrial oxidation or turnover in hair cells	Pickett et al., 2018
Tg[myo6b:mitoEos]	Mitochondrially localized photoconvertible fluorophore in hair cells	Pickett et al., 2018

Forward genetic screens have been particularly useful for identifying novel gene function in the zebrafish lateral line. One such gene identified using forward genetics is *pappaa*, which encodes pregnancy-associated plasma protein-aa (Wolman et al., 2015). Pappaa acts in the IGF1 signaling pathway and has recently been shown to regulate hair-cell mitochondrial function (Alassaf et al., 2019). *pappaa*^*p*170^ mutant zebrafish were more susceptible to neomycin-induced hair-cell death and had elevated ROS levels in their hair cells. In addition, *pappaa*^*p*170^ mutant hair cells had increased mitochondrial Ca^2+^, hyperpolarized ΔΨ_m_, and reduced expression of the mitochondrial antioxidant genes *gpx, sod1*, and *sod2*, all of which could contribute to increased ROS levels. Treatment with the ROS scavenger mitoTEMPO rescued *pappaa*^*p*170^ mutant susceptibility to neomycin-induced hair-cell death, suggesting that elevated ROS underlies the enhanced hair-cell death in *pappaa*^*p*170^ mutants. The study supports the utility of zebrafish forward genetic screens in identifying novel genes involved in mitochondrial function and hair-cell vulnerability.

Another study used CRISPR/Cas9 technology to delete the gene *mtu1* to study its function in hair cells (Zhang et al., 2018). This gene encodes a highly conserved mitochondrial tRNA modifying enzyme. In humans, deficient tRNA modification is associated with deafness (Wang et al., 2016). *mtu1*-deficient zebrafish had deficient thiolation of mitochondrial tRNA, as well as decreased levels of mitochondrial tRNA and mitochondrial proteins (Zhang et al., 2018). *mtu1*^−/−^ zebrafish also had deficient oxidative phosphorylation and reduced ATP. In the lateral line, *mtu1*^−/−^ zebrafish had fewer hair cells per neuromast. These results support a role for mitochondrial tRNA modification in deafness and demonstrate the value in using reverse genetics to study gene function in hair cells.

## Conclusion

The zebrafish lateral line is a valuable model system in which to study hair-cell mitochondria and offers unique tools such as the ability to visualize mitochondrial dynamics *in vivo*. Studies utilizing this system have shed light on the roles of mitochondria in calcium homeostasis and synapse regulation as well as supported roles of mitochondria in cell death pathways, particularly in response to ototoxic drugs like aminoglycosides. The strides made from zebrafish studies contribute to the understanding of hearing loss in humans and will lead to development of preventative or protective therapies in the future.

## Author Contributions

MH and LS wrote the manuscript. All authors contributed to the article and approved the submitted version.

## Conflict of Interest

The authors declare that the research was conducted in the absence of any commercial or financial relationships that could be construed as a potential conflict of interest.
